# *TCF7L2* gene associated postprandial triglyceride dysmetabolism- a novel mechanism for diabetes risk among Asian Indians

**DOI:** 10.3389/fendo.2022.973718

**Published:** 2022-10-03

**Authors:** Sri Venkata Madhu, Brijesh Kumar Mishra, Velmurugan Mannar, Mohd Aslam, Basudev Banerjee, Vivek Agrawal

**Affiliations:** University College of Medical Sciences, University of Delhi, Delhi, India

**Keywords:** *TCF7L2* gene, postprandial hypertriglyceridemia, type 2 diabetes mellitus, prediabetes, adipose tissue

## Abstract

**Aim:**

*TCF7L2* gene is believed to increase the risk of T2DM by its effects on insulin secretion. However, the exact mechanism of this enhanced risk is not clearly known. While *TCF7L2* gene has been shown to affect lipid metabolism, these effects have remained largely unexplored in the context of diabetes risk.

**Methods:**

Postprandial lipid responses to a standardized fat challenge test were performed in 620 Asian Indian subjects (310 with NGT and 310 with T2DM/prediabetes) and compared between the risk and wild genotypes of the rs7903146 *TCF7L2* gene. In 30 subjects scheduled to undergo abdominal surgery (10 each with NGT, Prediabetes and T2DM), adipocyte *TCF7L2* gene expression was also performed by real time qPCR and confirmed by protein expression in western blot.

**Results:**

T allele of rs7903146 *TCF7L2* gene was confirmed as the risk allele for *T2DM* (OR=1.8(1.2-2.74), p=0.005). TT+CT genotypes of rs7903146 *TCF7L2* gene showed significantly higher 4hrTg (p<0.01), TgAUC (p<0.01), peakTg (p<0.01) as well as higher postprandial plasma glucose (p=.006) levels and HOMA-IR (p=0.03) and significantly lower adiponectin levels (p=0.02) as compared to CC genotype. The expression of *TCF7L2* gene in VAT was 11-fold higher in prediabetes group as compared to NGT (P<0.01) and 5.7-fold higher in T2DM group as compared to NGT group(P=0.003) and was significantly associated with PPTg and glucose levels.

**Conclusion:**

There is significant PPTg dysmetabolism associated with the risk allele of rs7903146 polymorphism as well as adipocyte expression of *TCF7L2* gene. Significant upregulation of TCF7L2 gene expression in VAT that correlates with PPTg and glycaemia is also seen in Asian Indians with glucose intolerance. Modulation of PPTg metabolism by *TCF7L2* gene and the resultant *PPHTg* may be a novel mechanism that contributes to its diabetes risk in them.

## Introduction

Transcription factor 7 like 2 (*TCF7L2*) gene has been shown to have the strongest association with type 2 diabetes mellitus among all diabetogenic genes and this association has been replicated in all countries of the world including India (1–6). The risk of type 2 diabetes mellitus (T2DM) increases by 50% in the presence of the risk allele of *TCF7L2* gene at an individual level while the population attributable risk varies between 10-25% depending on the allele frequency (1, 7, 8). *TCF7L2* gene variant has also been shown to predict future diabetes risk in subjects with impaired glucose tolerance (9, 10). Despite this strong and consistent association, the exact mechanism of action by which this gene enhances diabetes risk is still unclear. The *TCF7L2* gene encodes a high mobility group (HMG) box-containing transcription factor, which is the effector of the canonical WNT signalling pathway that plays a key role in cell differentiation (11–13). *TCF7L2* protein also regulates the expression of several genes involved in cell cycle process including cyclin D1 and c-myc, which control the G1 to S phase transition (12, 14). It is generally believed that its effect on insulin secretion is the primary mechanism underlying its association with T2DM. Most studies have focused on the effects of *TCF7L2* gene on beta cell proliferation where it was found that altered expression of *TCF7L2* gene in beta cells of the pancreas results in impaired insulin secretion (9, 10, 15) Dominant-negative mutant of *TCF7L2* abolishes pro glucagon mRNA levels (16, 17). *TCF7L2* gene also affects GLP1 production and gluconeogenesis (18). Very few studies have explored its effects on adipogenesis and lipid metabolism as a possible mechanism for the *TCF7L2* gene associated risk of development of diabetes (19–22).

Postprandial hypertrglyceridaemia (PPHTg) may play an important role in the pathogenesis of diabetes (23–27). Recently, we have shown that PPHTg can predict the development of insulin resistance and diabetes (28). *TCF7L2* gene is known to upregulate the WNT signalling pathway in the adipose tissue and the resultant decrease in adipogenesis can lead to PPHTg (11, 13, 14, 16, 29). In an earlier study, variants of *TCF7L2* were found to modulate postprandial triglycerides levels in individuals with polyunsaturated fatty acid (PUFA) intakes of more than 7.36% (21). However, one of the studies conducted at our institute in subjects with normal glucose tolerance (NGT) did not show a significant difference in postprandial hypertriglyceridemia between the risk allele and wild type variants of this gene in first degree relatives of diabetes patients who had NGT and lower BMI (30). Decreased adipogenesis due to altered *TCF7L2* gene (11) function may result into impaired Tg trapping and PPHTg. The resultant overflow of triglycerides to ectopic sites such as liver and muscles may lead to lipotoxicity, insulin resistance and diabetes. Therefore, we aimed to study the polymorphisms of *TCF7L2* gene and its expression in subcutaneous and visceral adipose tissue to find out its association with postprandial hypertriglyceridaemia and risk of diabetes in subjects with varying degree of glucose tolerance.

## Methods

### Ethical considerations

The study was approved by Institutional Ethics Committee of University college of medical sciences and Guru Teg Bahadur Hospital. A written informed consent was obtained from all the subjects prior to the initiation of the study which was conducted in accordance with the principles of Declaration of Helsinki (31)

### Study population

It was a case control study conducted in 620 Asian Indians subjects (310 NGT, 310 subjects with glucose intolerance) between the age group of 20-60 years. The subjects with normal glucose tolerance, prediabetes and T2DM were identified on the basis of standard oral glucose tolerance tests as per the ADA criteria. Known diabetes subjects were recruited from Diabetic clinic of Department of Endocrinology. The genotype frequency of rs7903146 polymorphic form of *TCF7L2* gene in the literature was used to calculate the sample size required (2). The reported genotype frequency and the required number of subjects in each group to detect a significant difference in genotyping pattern using 2/3 chi-square with 5% alpha error and 90% power came out to be a minimum of 295 for each group. The sample size calculated to study the association between postprandial Tg parameters and genotypes of *TCF7L2* with 5% alpha error and 90% power came out to be 55 for each group based on existing literature. However, we have considered the higher sample size so that the objectives of this study can be met. Groups were matched for age, sex and BMI. Subjects with any chronic disease such as hyperlipidaemia inherited lipoprotein metabolism disorder, pancreatic disease, obstructive biliary disease or coronary heart disease, liver or kidney disease, hypothyroidism, Cushing’s syndrome, chronic diarrhoea, and malabsorption **a**ffecting lipid metabolism were excluded. Flow chart of study design is given in **Figue 1**.

**Figure 1:**
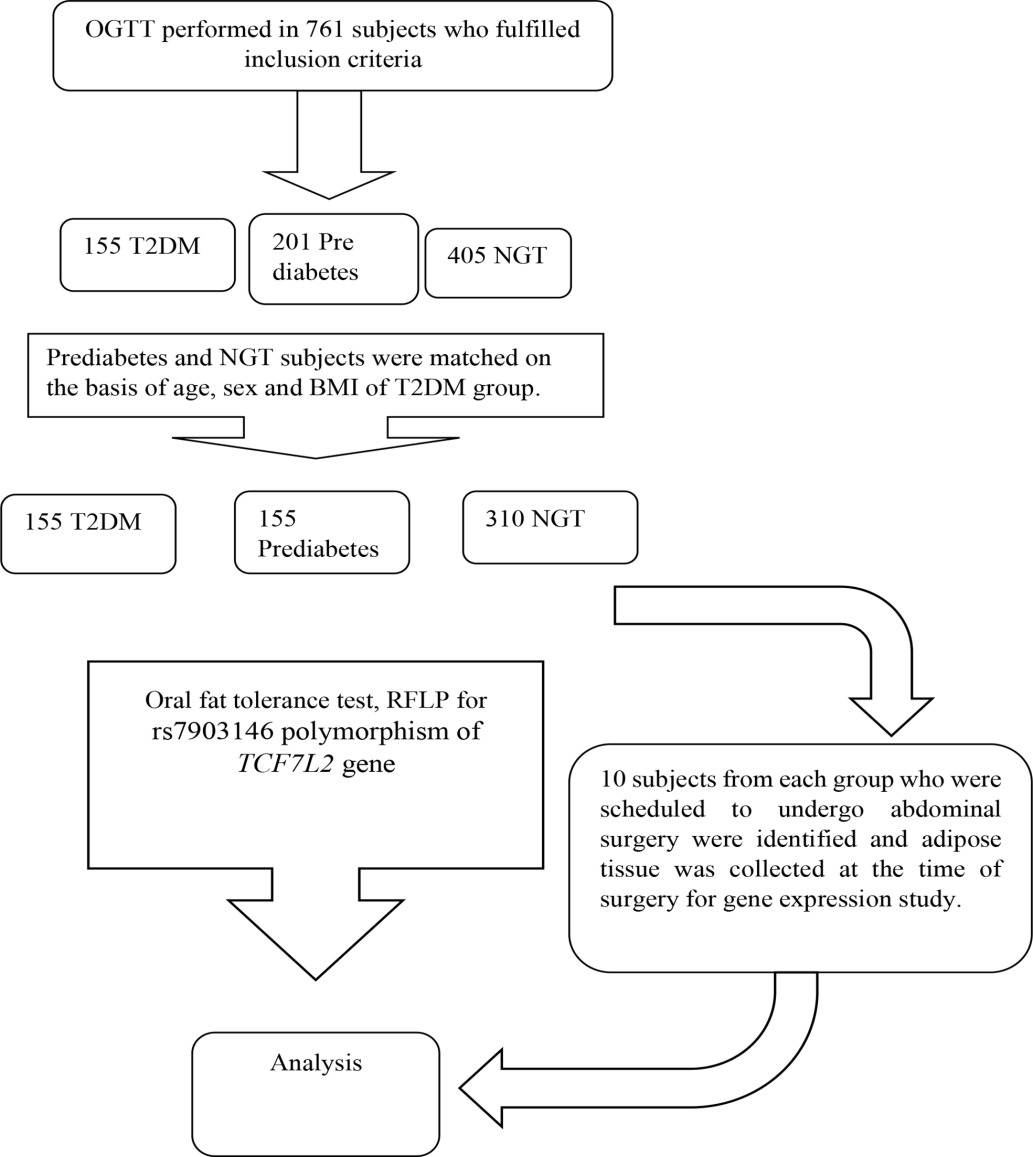
Flow chart of study.

### Fat challenge test and Biochemical estimations

A standardized 75 gm oral glucose tolerance test was performed in all the subjects to characterize them into different categories of glucose intolerance i.e., NGT and subjects with glucose intolerance on the basis of ADA criteria (32). Fasting blood samples was also collected for estimation of insulin levels. One week post Oral glucose tolerance test (OGTT), all subjects were called after overnight fasting for a standardized oral fat challenge test (33). Fasting blood samples were collected for the measurement of lipids, adiponectin and DNA extraction. A standardized fatty meal containing 729 kcal/m^2^ body surface area of calories, 62.5 gm of fat, 24.75 gm of carbohydrates, 5.3 gm of protein and 240 mg of cholesterol, that was made from whipped cream, banana and sugar was given to the subjects to be consumed over a period of 10 to 15 min. Blood samples were collected for estimation of lipids after the fat challenge 2 hourly upto 4 hours in all subjects and upto 8 hours in 2/3^rd^ of them.

Plasma glucose levels, Glucose Oxidase - Peroxidase (GOD POD method; Randox UK catalogue number: GL8308), serum insulin (IRMA method using ^125^I isotopes; Backmencoulter, catalouge no.-IM3210) and HbA_1_C (Biorad D-10 catalouge no: 2200101) were measured. Serum triglyceride, Total cholesterol and HDL were measured using Randox UK kits (Catalouge no; Tg: 120211, Tchol: 120194, HDL: CH2673) and adiponectin levels were estimated using Bio Vendor Elisa kits (Catalouge No. – RD194015200R). All the investigation procedures were initially standardized by repeatedly running the tests on same set of samples under a range of conditions. Homeostatic model assessment of insulin resistance (HOMA-IR) was calculated using fasting insulin and fasting plasma glucose levels.

### DNA extraction and genotyping

DNA was extracted from whole blood collected in EDTA tube by spin protocol using Qiagen kit (QIAamp DNA Mini kit, catalouge no. 51306) The rs7903146 polymorphism of *TCF7L2* gene was genotyped in 620 subjects using the polymerase chain reaction- restriction fragment length polymorphism method using following primers: forward primer 5’AAGAGAAGATTCCTTTTTAAATGGTG3’Reverse primer 3’CCTCATACGGCAATTAAATTATACA5’ (genei). A final reaction volume of 25 µl for the PCR was made which contained 100 ng of whole DNA, PCR master mix Thermo scientific #k0172 (0.05 U/µL Taq DNA polymerase, 4 mM Mgcl2, 0.4 mM of dNTP) and nuclease free water to make up the volume. The PCR was carried out on Biorad T100 model under following condition 95°C: 3 min 95°C: 30 sec 55°C:40 sec 72°C:45 sec and 72°C: 5 min. HPYCHIV restriction enzyme were used for the digestion of PCR product at 65 °C for 5 min. The digested products were analysed by electrophoresis on 2% agarose gel.

### Sequencing

We have further confirmed the Restriction fragment length polymorphism (RFLP) results for each genotype (TT, CC and CT) in a representative sample through sanger sequencing. Three samples of each genotype of rs7903146 polymorphic form of *TCF7L2*gene PCR product were randomly selected and sequenced. For sequencing PCR products were first purified with a column-based purification kit (Invitrogen™), and sequenced by the Sanger method using an Applied Biosystems 3130 Genetic Analyzer. The recognition sites of the TaaI enzyme within the sequence region were analysed with the Serial Cloner V2.5 program. The homology of DNA sequences to the published sequences was determined by using the BLAST platform on the national centre for biotechnology information (NCBI) website. The figures of the peak obtained for different genotypes are given in **Supplementary Figure 1**.

### Collection of biopsies and Gene expression studies

Thirty subjects (10 subjects each of NGT, Pre diabetes, and T2DM) who were to undergo elective appendicectomy, inguinal hernia repair or laparoscopic cholecystectomy for gall bladder stone removal were recruited for the gene expression study if they fulfilled the inclusion and exclusion criteria and were matched for age sex and BMI. Approximately 1 cm^2^ biopsies of subcutaneous adipose tissue (SAT) and visceral adipose tissue (VAT) were taken during surgery. The same was washed in physiological saline and immediately transferred in liquid nitrogen and carried to the biochemistry lab. Biopsies were homogenized and fixed in Trizol LS reagent (Invitrogen, Thermo scientific, catalogue no. 15596026) on the same day and were kept at −80°C until analysis. Chloroform-isopropanol method was used for the extraction of RNA. The purity of RNA sample was assessed by measuring the absorption at 230 nm, 260 nm and 280 nm using nano drop. The average yield of RNA in SAT was 384.07 ng/ul and in VAT was 1198.8 ng/ul and the integrity of RNA was checked by running the samples on 3% agarose gel. The ratio of 260:280 between 1.9-2.1 were considered for further analysis. cDNA was synthesized using iscript cDNA synthesis kit Biorad USA (catalogue no. 1708891).

*TCF7L2* gene expression in subcutaneous and visceral adipose tissue was quantified by quantitative real time polymerase chain reaction (qPCR) experiment conducted on CFX Connect TM Real-Time PCR Detection System (BioRad, USA). As housekeeping genes, β actin and GAPDH were used. All the *samples* were run in triplicate along with negative control. The results were calculated using the comparative 2^-∆∆CT^ method (Livak Method) manually (34). PCR amplification condition for *TCF7L2* gene was: denaturation at 94°C for 3 min followed by 40 cycles of denaturation for 30 s, annealing at 55°C for 30 s, extension at 72°C, and final extension at 72°C for 9 min using the forward primer 5’AAGAGAAGATTCCTTTTTAAATGGTG3’ Reverse primer 3’CCTCATACGGCAATTAAATTATACA5’. One µg of cDNA was used uniformly along with PCR master mix (SsoFast EvaGreen Supermix, Biorad US catalogue number 1725200) containing 0.4 mM of each dNTPs and 4 mM MgCl2. The length of *TCF7L2* amplicon was 267 bp and the mRNA variants were X4-XM011533842. The PCR efficiency was calculated from the slope.

RIPA lysis buffer (G Biosciences cat no. R159) was used to extract total protein from homogenized visceral adipose tissue which was then quantified by Bradford protein assay (Bedford, MA, USA). SDS PAGE (10%) was used to separate proteins which were transferred to PVDF membrane (Biorad cat no. 12-0177). The PVDF was further blocked using 5% non-fat dry milk in TBST. Primary antibody *TCF7L2* (1:700) (abbkine, cat no.-Abp52252-1) of rabbit anti goat source and anti-β-actin (abbkine, cat no.-Abp50593-1) (1:1000) and secondary antibody (abbkine, cat no.-Abp 21020-2) were used. The luminescence was recorded under Chemidoc (Thermo scientific ECL Imager, USA) which was further quantified by using Image J software.

### Statistical analysis

Differences between the clinical characteristics of the group were calculated by using unpaired t-test (for two groups) and one-way analysis of variance (for more than two groups). Comparisons of distribution of polymorphic forms between the groups were done by Chi-square test. Logistic regression analysis was performed to find out the odds ratio for disease risk associated with genotypes. The fold changes for expression of genes were calculated by using Livak method. Differences in gene expression between the groups were assessed with Mann Whitney test. Spearman correlation was performed to find the association of different variables with each other. Statistical analysis was performed using the software SPSS 20.0

## Results

Total number of study subjects were 620 (Subjects with normal glucose tolerance =310; Subjects with glucose Intolerance=310). Mean age of study subjects was 40.67 ± 8.92 years and mean BMI was 27.98 ± 4.89 kg/m^2^.

**Table 1** shows comparison of clinical and biochemical parameters between the study groups. All the groups were matched for age, sex and BMI shown by the absence of any significant difference of these parameters between the groups.

**Table 1:** Clinical and biochemical parameters of the study groups.

	Subjects with normal glucose tolerance (n = 310)	Subjects with glucose intolerance (n=310)	P value
Age (years)	40.58 ± 10.02	40.80 ± 8.35	0.13
Sex (male/female)	148/162	154/156	0.19
BMI (Kg/m2)	27.45 ± 4.55	28.66 ± 5.17	0.08
FPg (mg/dl)	90 ± 7.2	122 ± 59.94	<0.01
PPPg (mg/dl)	112 ± 16.13	197 ± 85.38	<0.01
Fasting insulin (IU/litre)	13.06 ± 5.91	13.18 ± 9.03	0.32
HOMA IR	2.74 ± 2.99	3.99 ± 3.11	<0.01
Adiponectin (µg/ml)	6.60 ± 4.47	5.38 ± 3.6	0.08

Statical analysis used: independent t-test.

It was observed that the T allele of rs7903146 polymorphic form of *TCF7L2* gene was significantly higher in subjects with Glucose Intolerance (44.2%) as compared to NGT groups (35.6%). There was a significant association of the T allele with glucose intolerance (OR=1.66(1.14-2.18) p=0.005) which indicates that T allele is the risk allele (**Table 2**). When subjects were re-analysed after further dividing those with glucose intolerance into prediabetes and T2DM, it was found that T allele remained significantly higher in the T2DM group when compared to those with NGT (OR=1.8(1.2-2.74) p=0.005) or prediabetes (OR=1.5(1.7-2.25) p=0.03). T allele remained higher in the prediabetes group when compared with those with NGT (OR=1.4(0.99-2.05) p=0.05). Further, T allele was also significantly associated with T2DM (**Table 3**). The genotypic distribution in our study was as per the Hardy- Weinberg equilibrium.

**Table 2:** - Genotypic and allelic frequencies and estimates of relative risks for the rs7903146 polymorphic form of TCF7L2 gene in subjects with and without glucose intolerance.

Genotypes and Alleles	Subjects with Normal Glucose Tolerance (n=310)	Subjects with Glucose Intolerance (n=310)	P value	OR T allele (TT+CT) vs CC OR (95% CI)	P value	OR C allele (CC+CT) vs TT OR (95% CI)	P value
CC	153 (49.35%)	129 (41.65%)	0.13	1.66 (1.14-2.18)	0.005	0.72 (0.50-0.98)	0.07
CT	93 (30%)	88 (28.38%)	0.71				
TT	64 (20.6%)	93 (30%)	0.009				
C allele	399 (64.4%)	346 (55.8%)	0.002				
T allele	221 (35.6%)	274 (44.2%)	0.002				

Statical analysis used, chi-square.

**Table 3:** - Genotypic and allelic frequencies and estimates of relative risks for the rs7903146 polymorphic form of TCF7L2 gene in subjects with normal glucose tolerance, Prediabetes and Diabetes.

Genotypes and Alleles	NGT(n=310)	Prediabetes(n=155)	T2DM(n=155)	P value	ORTT+CT vs CCOR (95% CI)	P value	ORCC+CT vs TTOR (95% CI)	P value
CC	153(49.35%)	72 (46.5%)	57 (36.7%)	a = 0.55 b= 0.1 c=0.01	T2DM vs NGT1.8 (1.2-2.74)	0.005	T2DM vs NGT0.45 (0.29-0.70)	0.78
CT	93 (30%)	42 (27.1%)	46 (29.7%)	a =0.91 b= 0.7 c= 0.48	PD vs NGT1.4 (0.99-2.05)	0.05	PD vs NGT0.78 (0.51-1.17)	0.09
TT	64 (20.6%)	41 (26.4%)	52 (33.5%)	a = 0.19, b= 0.21, c= 0.003				
C allele	399 (64.4%)	186 (60%)	160 (51.60%)	a = 0.7 b= 0.18 c= 0.03	T2DM vs PD 1.5 (1.17-2.55)	0.03	T2DM vs PD 0.68 (0.45-1.07)	0.10
T allele	221 (35.6%)	124 (40%)	150 (49.40%)	a = 0.7 b= 0.18 c= 0.03				

*a= NGT vs Prediabetes, b=Prediabetes vs T2DM, c=NGT vs T2DM.

*NGT, normal glucose tolerance; PD, prediabetes statical analysis used: chi-square.

**Figure 2:**
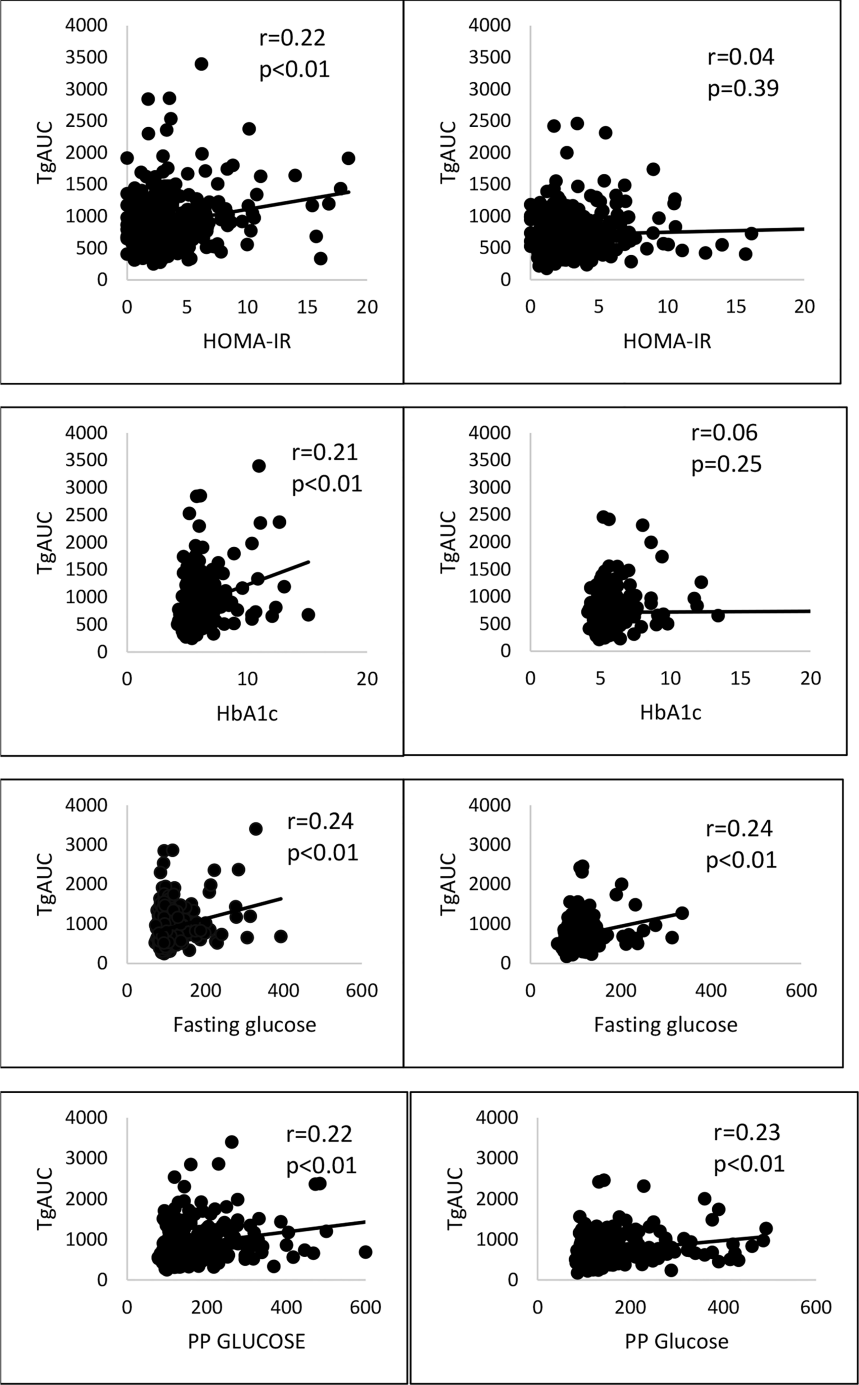
Correlation of risk and wild genotypes of rs7903146 polymorphic form of TCF7L2 gene with TgAUC, glycaemic and insulin resistance parameters. PP glucose, postprandial plasma glucose; TgAUC, triglyceride area under curve; HOMA-IR , Homeostatic model assessment of insulin resistance; statical analysis used: Pearson correlation.

Fat challenge test was carried out until 4 hours in all 620 subjects and lipid samples were collected till 8 hours in 406 subjects of them. Postprandial triglycerides (PPTg) levels and glycaemic parameters were compared between risk genotype and wild genotype of rs7903146 polymorphic form of *TCF7L2* gene. It was found that TT+CT genotypes (T allele) of rs7903146 polymorphic form of *TCF7L2* gene showed significantly higher Tg 4hr (p<0.01), TgAUC (p<0.01) and peakTg (p<0.01) levels as well as higher postprandial plasma glucose levels (p=0.006) and HOMA-IR(p=0.03), and significantly lower adiponectin levels(p=0.02) as compared to CC genotype (**Table 4**). **Figure 2** shows that there was a significant positive correlation of TgAUC with HOMA-IR (r=0.22, p<0.01) and HbA1c (r=0.21, p<0.01) in risk genotype subjects but not in subjects with wild genotype. However, fasting plasma glucose and postprandial plasma glucose correlated significantly with TgAUC in both the groups.

**Table 4:** - Comparison of postprandial triglyceride, glycaemic and insulin resistance parameters between Risk and wild type genotypes of rs7903146 polymorphic form of TCF7L2 gene in all the subjects.

	Risk genotype (TT+CT) (n=318)	Wild genotype (CC) (n=302)	P value
Postprandial Tg parameters			
TgAUC (4hour)	904 ± 453	714 ± 331	<0.01
Tg 4hr (mg/dl)	332 ± 177	261 ± 135	<0.01
TgAUC (8hour) (R=183, W=223)	2346 ± 1014	1798 ± 331	<0.01
PeakTg(mg/dl)(8 hour) (R=183, W=223)	903 ± 176	709 ± 136	<0.01
Glycemic parameters			
FPg (mg/dl)	112 ± 42	105 ± 32	0.09
PPPg (mg/dl)	171 ± 80	153 ± 74	0.006
Insulin resistance parameters			
Homa IR	3.67 ± 2.40	2.77 ± 1.73	0.03
Adiponectin(µg/ml)	5.20 ± 3.68	6.72 ± 4.38	0.02

R, Risk genotype; W,wild genotype; FPg, fasting plasma glucose; PPPg, postprandial plasma glucose; Tg, Triglycerides; PPTg, postprandial triglycerides; TgAUC, triglyceride area under curve; HOMA-IR , Homeostatic model assessment of insulin resistance statical analysis used: independent t-test.

**Figure 3:**
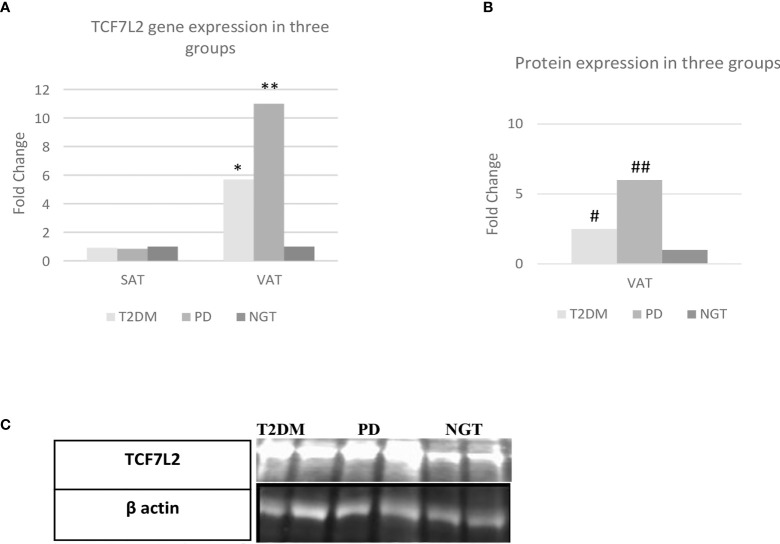
TCF7L2 gene/protein expression in the three study groups. VAT= visceral adipose tissue, SAT= subcutaneous adipose tissue, NGT, normal glucose tolerance; PD, prediabetes; T2DM, type 2 diabetes mellitus; N=30; statical analysis: one way anova; **= Prediabetes vs NGT (p=≤0.01); *= T2DM vs NGT (p=≤0.01); # = Prediabetes vs NGT (p=≤0.01); ## = T2DM vs NGT (p=≤0.01). **(A)** Fold Change of TCF7L2 gene expression between the three groups. **(B)** Fold Change of TCF7L2 protein expression between the three groups. **(C)** Representative western blot image.

Adipocyte expression of *TCF7L2* gene in VAT and SAT is shown in **Figure 3**. The expression of *TCF7L2* gene in VAT was 11-fold higher in prediabetes group as compared to NGT (P<0.01) and 5.7-fold higher in T2DM group as compared to NGT group (P<0.01). Protein expression of *TCF7L2* gene in VAT by western blot technique confirmed the findings of gene expression viz. 6-fold higher *TCF7L2* expression in Prediabetes group and 2.5-fold higher expression in T2DM group as compared to NGT group as shown in **Figure 3**.

There was no significant difference in the expression of *TCF7L2* gene between the three groups in SAT.

**Figure 4:**
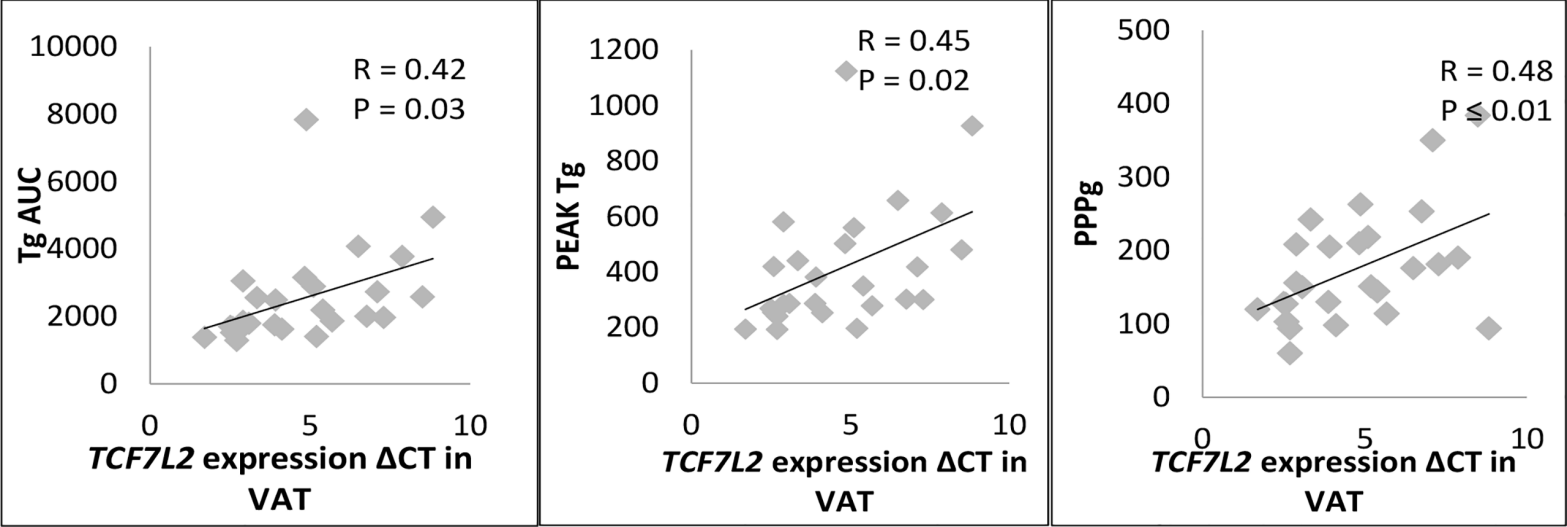
Correlation of TCF7L2 gene expression in VAT with PPTg and Glycaemic parameters. PPg, postprandial plasma glucose; Tg, Triglycerides; TgAUC, triglyceride area under curve; N=30 ; statical analysis: Pearson correlation.

There was a significant correlation of expression of *TCF7L2* gene in visceral adipose tissue with TgAUC (p<0.03), PeakTg (p<0.02) and postprandial plasma glucose levels (p<0.01) (**Figure 4**). However, there was no correlation of expression of *TCF7L2* gene in visceral adipose tissue with fasting plasma glucose (p =0.15), HOMA-IR (p =0.12) or serum adiponectin levels (p =0.3).Also, there was no significant correlation of expression of *TCF7L2* gene in subcutaneous adipose tissue with any of the PPTg, glycaemic or insulin resistance parameters.

## Discussion

Our study found that individuals with TT/CT genotypes of rs7903146 polymorphism showed significantly higher levels of postprandial triglycerides, fasting and postprandial plasma glucose, and HOMA-IR and significantly lower serum adiponectin levels as compared to CC genotype. Those with TT/CT genotypes were at a significantly higher risk of having glucose intolerance as compared to CC genotypes. Adipocyte expression of *TCF7L2* gene in visceral adipose tissue was significantly higher in those with glucose intolerance as compared to NGT. Furthermore, there was a significant positive correlation between *TCF7L2* gene expression in visceral adipose tissue and PPTg parameters such as TgAUC, PeakTg as well as postprandial plasma glucose.

The strength of the present study is that it not only seeks to explore the association of the rs7903146 polymorphic form of *TCF7L2* gene with altered postprandial triglycerides metabolism but also examines for the first time whether altered adipocyte expression of this gene could also be associated with significant postprandial hypertriglyceridemia and insulin resistance.

The association of rs7903146 polymorphic form of *TCF7L2* gene with enhanced risk of diabetes has been consistently demonstrated from different populations (4, 35, 36) in the world including Asian Indians (2, 3, 37). However, the underlying mechanisms are not fully understood. It is believed that these effects are secondary to an impairment in insulin secretion (10, 15). The role of *TCF7L2* gene in modulating lipid metabolism is a less well studied area although these are mentioned in literature (19–22, 38, 39)

Our previous studies showed that postprandial hypertriglyceridemia is seen early in the natural history of diabetes and also in first degree relatives of subjects with prediabetes (33) and could lead to the development of prediabetes and diabetes (40). This prompted us to ascertain if postprandial triglycerides dysmetabolism could be an important and novel mechanism by which *TCF7L2* associated risk of diabetes is mediated.

The current study demonstrated the association of TT/CT genotypes of the rs7903146 polymorphic form of *TCF7L2* gene with PPTg levels in a wide range of subjects from both sexes aged between 20-60 years and included those with all classes of glucose tolerance. This would suggest a consistent and robust association between *TCF7L2* gene with PPTg metabolism. The significantly higher PPTg levels in subjects with the T allele who also displayed greater insulin resistance and a higher risk of glucose intolerance clearly indicate that the risk allele of this gene is associated with PPTg dysmetabolism. After thorough research of the literature, only 5-6 studies could be identified which investigated the influence of *TCF7L2* gene with respect to postprandial lipemia (19–21, 30, 38, 41). Association of T allele of rs7903146 polymorphic form of *TCF7L2* gene and postprandial hypertriglyceridemia was reported by Martinez et al. in young and elder healthy males (19). However, these researchers could not demonstrate altered PPTg metabolism in subjects with metabolic syndrome. In another study, Musso G et al. demonstrated that TT/CT rs7903146 polymorphic form of *TCF7L2* gene was involved in modulation of postprandial lipids and displayed higher postprandial lipoprotein levels in patients with NASH (41). Also, healthy subjects with T allele with PUFA intake of more than 7.36% were shown to have higher VLDL concentrations and postprandial triglyceride lipoproteins (21). However, there was no association of postprandial triglyceride levels with risk allele of *TCF7L2* gene in healthy first degree relatives of diabetes patients who had NGT and a BMI < 25 Kg/m^2^ (22). Each of these studies represented specific groups of subjects mostly healthy individuals without diabetes or prediabetes unlike in our study which included subjects with varying degree of glucose tolerance.

Logistic regression analysis revealed that the risk of T2DM was significantly higher in subjects with TT genotype of rs7903146 polymorphic form of *TCF7L2* gene as compared to CC genotype which confirms T allele as the risk allele for this polymorphism as reported earlier (2, 3, 37). TT/CT genotypes of the rs7903146 polymorphic form of *TCF7L2* gene are also associated with higher HOMA-IR and lower serum adiponectin levels both of which are markers of higher insulin resistance. Similar results were shown in an earlier study (42). Taken together, our findings with respect to rs7903146 polymorphism indicate that postprandial dysmetabolism could be an important contributor to *TCF7L2* associated insulin resistance.

Whether *TCF7L2* gene associated postprandial lipid dysmetabolism leads to insulin resistance or *TCF7L2* associated insulin resistance results is postprandial lipid dysmetabolism cannot be said with certainty from the current study. Association of the risk allele of *TCF7L2* gene with insulin resistance (43) and post meal insulin resistance (44) has been reported before. However, when read with previous studies that have suggested that postprandial lipemia is an early phenomenon in diabetes, prediabetes (27), first degree relatives of T2DM (24, 27) as well as our previous study in animals (28), it would appear likely that it is TCF7L2 gene associated PPHTg that results in insulin resistance and not vice versa.

We also found a significant positive correlation between expression of *TCF7L2* gene in visceral adipose tissue and PPTg parameters such as TgAUC and PeakTg levels in subjects who had varying levels of glucose intolerance. This is the first demonstration of a significant association between *TCF7L2* expression in visceral adipose tissue and PPTg dysmetabolism confirming the findings from our polymorphism studies and points to an important role of *TCF7L2* gene in postprandial hypertriglyceridemia. We did not find any study in the literature regarding *TCF7L2* gene expression in adipose tissue in the context of postprandial lipemia.

We found a significantly higher expression of *TCF7L2* gene in VAT in patients with T2DM while there was no significant change in SAT. This finding in VAT is both novel as well as interesting and may be related to the unique pattern of body fat distribution described in Asian Indians. Results of previous studies on adipose tissue *TCF7L2* gene expression in T2DM and impaired glucose metabolism have been conflicting and have reported both upregulation (45) as well as downregulation (46) of *TCF7L2* gene in adipose tissue. Few studies reported no significant change in *TCF7L2* expression also (47) for expression studies (46, 48–50). The increased adipose tissue expression of *TCF7L2* gene in our study in subjects with glucose intolerance may reflect the known phenotypic differences in adipose tissue morphology and distribution in both these populations. It is well known that Asian Indians have significantly higher VAT and significantly lower SAT when compared to white Caucasians (51–53). This, phenotype which has been described as the “thin fat” Indian phenotype, is associated with (54) normal body weight in many cases, and is believed to be a form of partial lipodystrophy that facilitates ectopic lipid accumulation in visceral adipose depots. Central obesity and higher VAT depots are believed to be central to the development of IR and T2DM in south Asians including Asian Indians (51).

Recent genetic and transcriptomic studies using gene set enrichment analysis from India have shown that Adipocyte is a key player in the pathogenesis of insulin resistance and T2DM in Indians (55–57). Asian Indians showed differentially expressed lipodystrophy genes in peripheral subcutaneous tissue of type 2 diabetic patients (55) significantly greater expression of several genes affecting adipogenesis and inflammation, particularly in VAT and visceral adipocyte hypertrophy in diabetic subjects compared to controls whereas in SAT the difference in adipocyte size was not significant (55). Inadequate activation of adipogenesis results in a lower capacity of SAT to store fat and any response to chronic overnutrition in this scenario is by adipocyte hypertrophy in SAT and VAT (58). It has therefore been suggested that genetic predisposition for T2DM in south Asians is associated with restricted adipogenesis and hypertrophic obesity (59). It is well known that *TCF7L2* gene activates the WNT pathway through the effects of beta-catenin in the visceral adipose tissue thereby inhibiting adipogenesis (12, 60). It is therefore possible that the *TCF7L2-related* genetic predisposition to T2DM in Asian Indians is at least partly mediated by its effects on the WNT pathway. The finding of increased *TCF7L2* expression in VAT in our study supports this hypothesis. However, it will not be possible from this study to say that it is only the overexpression of *TCF7L2* gene in visceral adipose tissue that results in insulin resistance. It could also be possible that insulin resistance in these subjects upregulates this gene. Only longitudinal cohort studies can answer this with clarity.

Recent evidence reveals a more complex regulatory role for *TCF7L2* gene and its adipose tissue expression in mediating WNT pathway related metabolic effects that are associated with Insulin resistance and T2DM. Chen et al. have clearly demonstrated that there could be important differences depending on whether *TCF7L2* expression is in preadipocytes or in mature adipocyte. In preadipocytes with high levels of beta-catenin, *TCF7L2* overexpression is associated with greater beta-catenin binding that facilitates WNT activation and inhibition of adipogenesis whereas in mature adipocytes, with low levels of beta-catenin, it is TCF7L2 gene downregulation that activates WNT pathway leading to similar metabolic effects such as adipocyte hypertrophy, increased fat deposition in the liver and insulin resistance (11). It is very well possible that in Caucasians, who have a much higher subcutaneous fat depot (SAT) and an abundance of mature adipocytes, it is the down regulation of *TCF7L2* gene in mature adipocytes that plays a dominant role in causing insulin resistance and glucose intolerance. On the contrary, in Asian Indians, the paucity of mature adipocyte (61) secondary to the partial lipodystrophic phenotype would make any effect of *TCF7L2* gene under expression in these adipocytes far less significant. Hence, at least in Asian Indians the overexpression of *TCF7L2* gene in preadipocytes is likely to be having a dominant role in the development of adipocyte hypertrophy and all its consequences leading to T2DM particularly in the face of chronic high energy intake. Clearly, more research is required to understand the precise role of *TCF7L2* and its adipose tissue expression in adipogenesis and adipocyte function in relation to glucose intolerance and T2DM.

Our study revealed a significant association of *TCF7L2* expression in VAT with all PPTg and glycaemic parameters. Activation of the canonical WNT pathway in VAT secondary to upregulation of *TCF7L2* gene can also result in impairment of Tg trapping by the visceral adipose tissue and consequent PPHTg. The resultant lipid spill over and ectopic deposition of fat in liver, muscles and pancreas can lead to insulin resistance and type 2 diabetes (49, 62). We propose that dysregulation of *TCF7L2* gene can result in postprandial hypertriglyceridemia through its actions on the WNT pathway in VAT and could thereby contribute to the associated diabetes risk. This could be a novel mechanism by which *TCF2L2* gene enhances the risk of T2DM

### Limitations

There are a few limitations of this study. In the polymorphism study, only rs7903146 SNP of *TCF7L2* gene was explored as it was most commonly associated with T2DM. Examining other SNPs could have provided additional information on the association studied. We could analyze only representative samples by Sanger sequencing due to cost limitations; it would have been better to perform sequencing in all the samples. We did not use microarray for gene expression experiments which could have further enhanced the accuracy of the results. However real-time PCR is considered a valid method to perform gene expression studies. Lastly our findings need to be validated through prospective cohort studies.

## Conclusion

There is significant PPTg dysmetabolism associated with the risk allele of rs7903146 polymorphism as well as adipocyte expression of *TCF7L2* gene. Significant upregulation of *TCF7L2* gene expression in VAT that correlates with PPTg and glycaemia is also seen in Asian Indians with glucose intolerance. Modulation of PPTg metabolism by *TCF7L2* gene and the resultant PPHTg may be a novel mechanism that contributes to its diabetes risk in them.

## Data availability statement

The original contributions presented in the study are publicly available. This data can be found here: Gene Bank data repository with the accession number 2622729.

## Ethics statement

The studies involving human participants were reviewed and approved by Institutional Ethics Committee of University college of medical sciences & Guru Teg Bahadur Hospital. Written informed consent to participate in this study was provided by the participants’ legal guardian/next of kin.

## Author contributions

BM: Contributed in conception and design, acquisition ofdata, data analysis, interpretation of data, drafting the article.VM: acquisition of data, writing work. MA: acquisition of data,data analysis. BB: contributed in conception and design, revising article. VA: contributed in conception and design, acquisition of data. SM: Contributed in conception and design, data analysis,interpretation of data, revising article and final approval of theversion to be published.

## Funding

This work was supported by Indian Council of Medical Research, New Delhi. (Grant No. 5/4/5-3/Diab-16-NCD-II).

## Conflict of interest

The authors declare that the research was conducted in the absence of any commercial or financial relationships that could be construed as a potential conflict of interest.

## Publisher’s note

All claims expressed in this article are solely those of the authors and do not necessarily represent those of their affiliated organizations, or those of the publisher, the editors and the reviewers. Any product that may be evaluated in this article, or claim that may be made by its manufacturer, is not guaranteed or endorsed by the publisher.
