# Supplementary figures and images for: TCF7L2 gene associated postprandial triglyceride dysmetabolism- a novel mechanism for diabetes risk among Asian Indians
Source: Front Endocrinol (Lausanne). 2022 Oct 3;13:973718. doi: 10.3389/fendo.2022.973718 (PMC9573951; doi:10.3389/fendo.2022.973718)

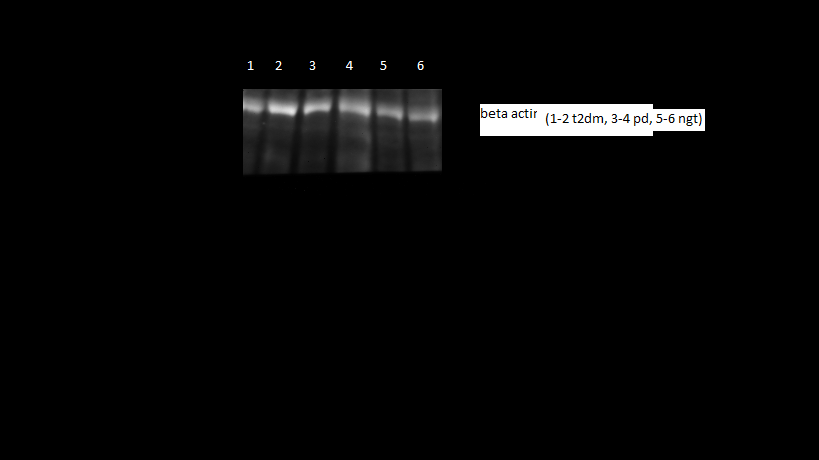

Supplement: Supplementary file 1 [file Image_1.png]

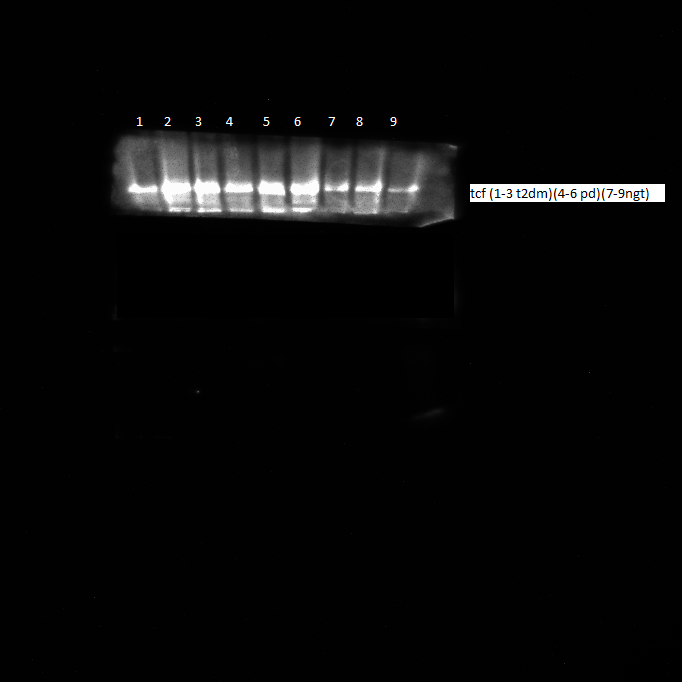

Supplement: Supplementary file 2 [file Image_2.tif]

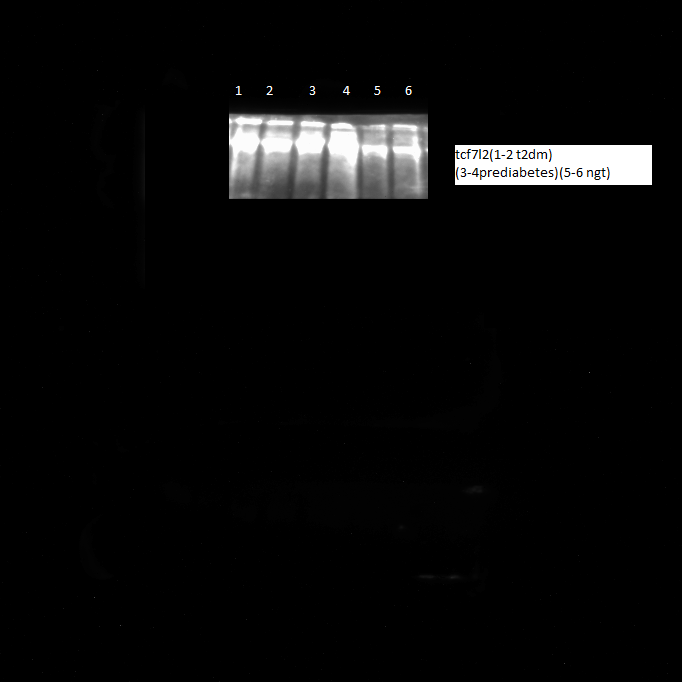

Supplement: Supplementary file 3 [file Image_3.tif]

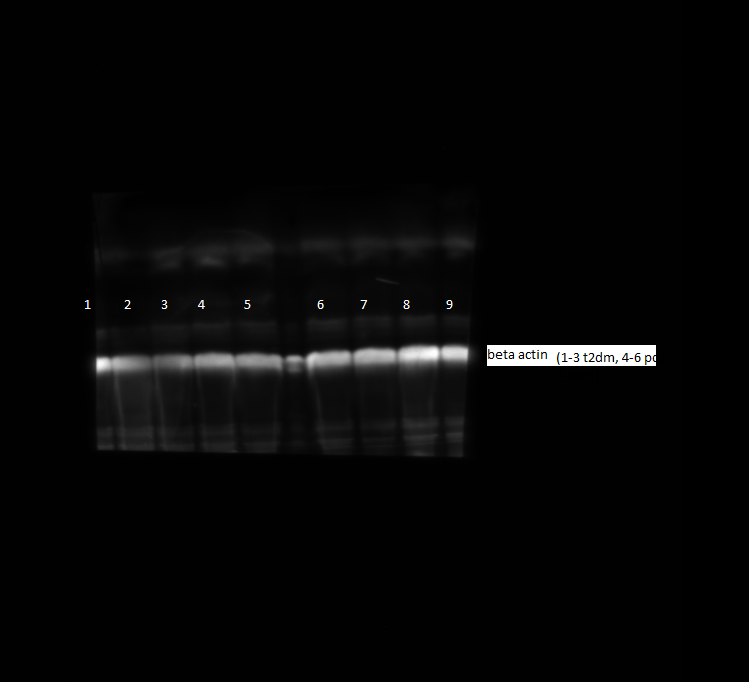

Supplement: Supplementary file 4 [file Image_4.tif]
